# Experiences with a violence and mental health safety protocol for a randomized controlled trial to support youth living with HIV

**DOI:** 10.1186/s41256-021-00224-0

**Published:** 2021-10-15

**Authors:** Katherine G. Merrill, Jonathan K. Mwansa, Sam Miti, Virginia M. Burke, Elizabeth A. Abrams, Christiana Frimpong, Michele R. Decker, Jacquelyn C. Campbell, Julie A. Denison

**Affiliations:** 1 Department of International Health, Johns Hopkins Bloomberg School of Public Health, 615 N. Wolfe Street, Baltimore, MD 21205 USA; 2 Arthur Davison Children’s Hospital, Ndola, Zambia; 3 Department of Population, Family and Reproductive Health, Johns Hopkins Bloomberg School of Public Health, Baltimore, MD USA; 4 Department of Community-Public Health, Johns Hopkins School of Nursing, Baltimore, MD USA; 5 Present Address: Department of Medicine, University of Illinois at Chicago, Chicago, IL USA

**Keywords:** Safety protocol, Referral, Violence, Mental health, HIV, Youth, Zambia

## Abstract

**Background:**

Safety protocols are an essential component of studies addressing violence and mental health but are rarely described in the published literature from Sub-Saharan Africa. We designed and implemented a safety protocol within Project YES! (Youth Engaging for Success), which enrolled 276 youth living with HIV (ages 15–24 years) in a randomized controlled trial of a peer-mentoring intervention across four HIV clinics in Ndola, Zambia.

**Methods:**

Youth who reported severe violence and/or suicidal thoughts on research surveys or during meetings with youth peer mentors (YPM) were referred to designated healthcare providers (HCP). We explored experiences with the safety protocol using: a) monitoring data of referrals, and b) in-depth interviews with youth (n = 82), HCP (n = 10), YPM (n = 8), and staff (n = 6). Descriptive statistics were generated and thematic analysis of coded transcripts and written memos performed.

**Results:**

Nearly half of youth enrolled (48% of females, 41% of males) were referred to a HCP at least once. The first referral was most often for sexual violence (35%) and/or suicidal ideation/depression (29%). All referred youth aged 15–17 years and over 80% of referred youth aged 18 + agreed to see a HCP. HCP referred 15% for additional services outside the clinic. Twenty-nine youth, all HCP, all YPM, and all staff interviewed discussed the safety protocol. Most youth felt “encouraged,” “helped,” “unburdened,” and “relieved” by their meetings with HCP; some expressed concerns about meeting with HCP. The safety protocol helped HCP recognize the need to integrate care for violence and mental health with medication adherence support. HCP, YPM, and study staff raised implementation challenges, including youth choosing not to open up to HCP, time and resource constraints, deficiencies in HCP training, and stigma and cultural norms inhibiting referrals outside the clinic for emotional trauma and mental health.

**Conclusions:**

Implementing a safety protocol within an HIV clinic-based research study is possible and beneficial for youth and HCP alike. Implementation challenges underscore that HCP in Zambia work in over-stretched healthcare systems. Innovative strategies must address deficiencies in training and resources within HIV clinics and gaps in coordination across services to meet the overwhelming need for violence and mental health services among youth living with HIV.

**Supplementary Information:**

The online version contains supplementary material available at 10.1186/s41256-021-00224-0.

## Introduction

Adolescents and young adults face high levels of violence victimization in Sub-Saharan Africa (SSA), with the prevalence of physical, emotional, or sexual violence ranging from about 30 to 50 percent in some African settings [1, 2]. The prevalence of mental health problems in this population is also high. Roughly 20 to 30 percent of adolescents in SSA experience suicidal ideation, depression, and anxiety [3]. Violence victimization and mental health problems are related with each other and intersect with other developmental and health risks among youth, including increased substance use, lower educational achievement, and poorer sexual and reproductive health [4–6]. Among youth living with HIV, experiences of violence and mental health problems have been associated with negative HIV outcomes, including incomplete antiretroviral therapy (ART) adherence and virologic failure [7–10].

With the expansion of violence and mental health research has come a growing body of evidence on ethical and methodological considerations. Adhering to the fundamental ethical principles of respect for persons, beneficence, and justice is critical to protecting the safety of study participants and facilitating quality data [11]. Guidelines have been developed to promote safety, confidentiality, training, and informed consent in studies of violence [11, 12]. Recommendations target specific study populations, including children [13, 14], adolescents [15], trafficked women [16], women in conflict settings [17], and women with disabilities [18]. However, we identified few studies describing safety protocol implementation experiences in SSA for violence [19] or mental health [20], which is critical for building consensus around best practices for safety planning in these research areas [21]. Moreover, we found no guidance on researching violence and mental health among youth living with HIV in the region. In addition to the violence and mental health symptoms they commonly experience [7–9, 22], these youth are vulnerable due to the high levels of stigma and discrimination associated with HIV infection [23–25] and their developmental stage, which can make them prone to impulsivity and risky behavior [26].

We designed and implemented a safety protocol within the Project YES! (Youth Engaging for Success) randomized controlled trial (RCT) [27, 28]. The RCT assessed the impact of a peer mentoring intervention on viral suppression, ART adherence, and self-stigma among youth living with HIV. Youth were consecutively recruited and enrolled from four HIV clinics (a children’s hospital, an adult hospital, and two primary health facilities) in Ndola, Zambia [27]. The intervention consisted of individual and group meetings over six months with a youth peer mentor (YPM) who had successfully transitioned to HIV self-management. Youth who reporte d experiences of severe violence and/or suicidal thoughts during the research surveys (at baseline, 6 months post, and 12 months post), in their YPM meetings, or at another time during the study, were referred to designated healthcare providers (HCP) at the HIV clinics. In this analysis, we examine experiences with the safety protocol using quantitative descriptive statistics and qualitative in-depth interviews (IDI) with youth participants, HCP, YPM, and study staff. We aim to: (a) characterize youth’s first referrals, and (b) explore successes and challenges with the protocol’s implementation.

## Methods

### Study design

We employed a convergent, parallel mixed-methods design [29], with quantitative and qualitative data collected concurrently, analyzed independently, and interpreted jointly. Quantitative process data were meant to generate summary information on the numbers and types of referrals made. Qualitative IDI data were meant to facilitate a multidimensional view of the protocol’s implementation by capturing experiences, opinions, and perspectives from the youth themselves and those administering the safety protocol (i.e., HCP, YPM, and study staff). Data from each method was equally considered when triangulating the study findings.

### Safety protocol development

**Fig. 1:**
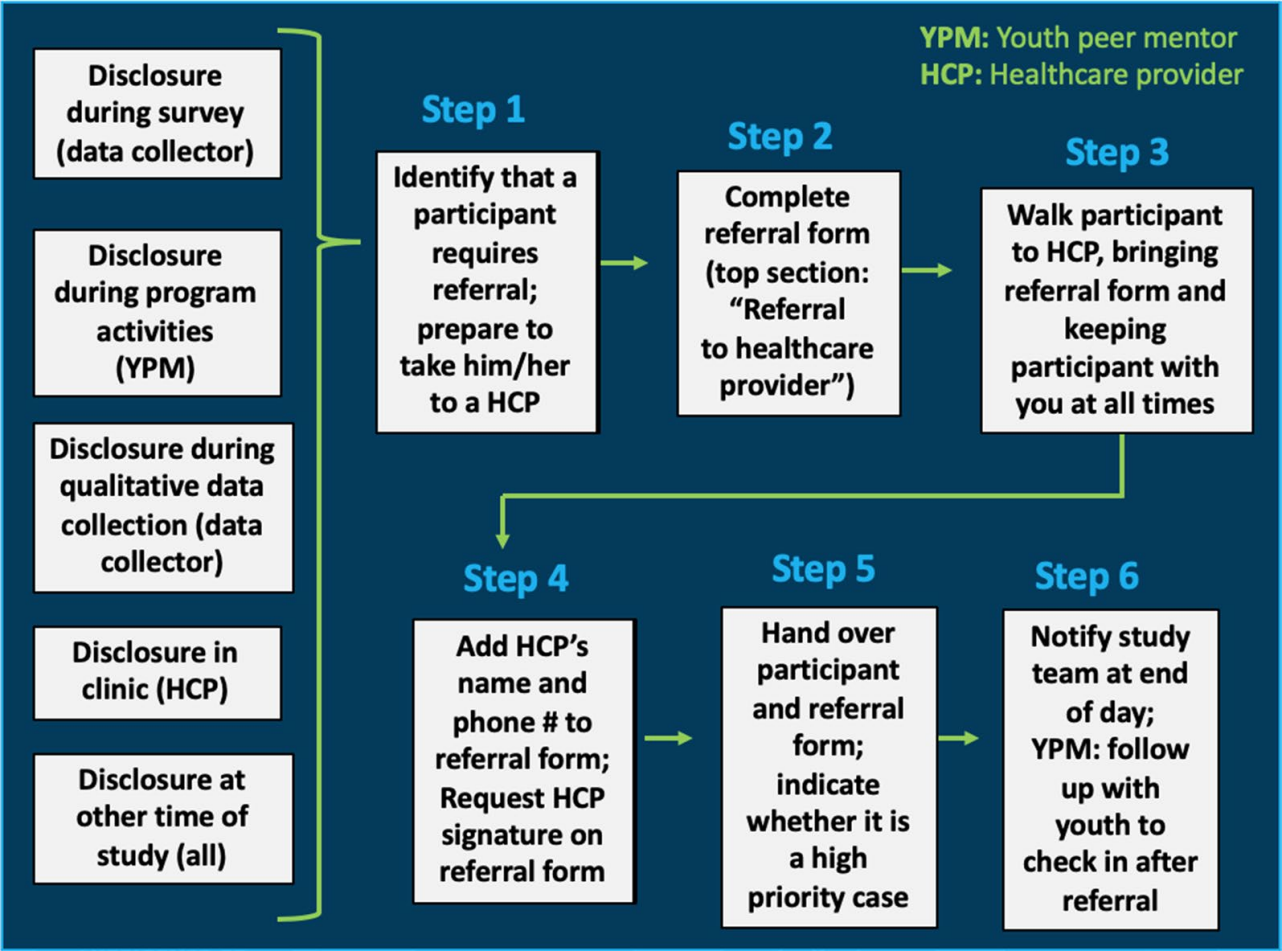
Summary of referral procedures for staff and youth peer mentors

To create a safety protocol, we consulted recommendations from the World Health Organization [12] and safety protocol experiences from other settings [19, 20, 30]. We also sought and incorporated feedback from HCP at each participating clinic. The protocol provided step-by-step guidance for addressing reports of violence and/or suicidal ideation (Fig. 1 and Additional File 1). When an issue was identified, YPM or study staff completed a form documenting the participant’s connection with the designated HCP (i.e., a “referral”). HCP handled cases according to clinical practice, local policy, and Zambian law. Where appropriate, HCP referred participants for services outside of the clinic (i.e., an “external referral”). We provided initial and ongoing safety protocol training for HCP, YPM, and study staff, in line with ethical guidelines [11].

Several adjustments were made throughout implementation to maintain our ethical obligations beyond the most basic “duty of care” [21]. At the request of HCP, Project YES! developed “community maps,” with resources on where youth could be referred outside of the clinic. To help facilitate external referrals, Project YES! provided transport and YPM accompanied youth who wanted it. Three months into the project, it became clear that youth often did not wish to discuss issues with the HCP at their clinic. In response, Project YES! hired and trained a mental health nurse on study procedures to be based at the children’s hospital but remain independent from the four participating HIV clinics.

### Data collection procedures

Referral forms tracked key information about the referral (e.g. date, role of person referring, etc.). HCP recorded notes about the meetings held and external referrals made in notebooks. Referral forms and HCP notebooks were stored under lock and key at each clinic. Information was regularly input into a secure, customized Excel database.

Questions about experiences with the safety protocol were integrated into semi-structured IDI with 82 youth participants, 10 HCP, and 8 YPM. Two groups of youth participants were sampled. Forty-one youth (22 female, 19 male) were interviewed about their experiences with Project YES!. These youth were purposively sampled from the intervention arm to achieve diversity in sex, age, change in virologic results between baseline and 6-month follow-up, and clinic. An additional 41 youth (24 female, 17 male) were interviewed about their experiences with violence victimization and HIV self-management. These youth were purposively sampled from both study arms to achieve maximum variation in their experiences of violence (moderate or severe), virologic results (failure or not), sex (male or female), and age group (15–19 or 20–24 years), using baseline survey data [31]. Youth were asked to describe what happened when they were asked to see a HCP and how they felt about the steps taken. These IDI were conducted by three Zambian interviewers who were not involved in the Project YES! intervention and had previous research experience with youth living with HIV. Prior to the project, they completed nine days of training, which included content on the study’s safety protocol. Interviewers and youth were matched by sex, and IDI were conducted in participants’ preferred language (English or Bemba).

All HCP (7 female, 3 male) and YPM (4 female, 4 male) were invited and agreed to participate in IDI (see further details elsewhere [32, 33]). Additionally, 6 IDI were held with consenting study staff involved in implementing the safety protocol (2 female, 4 male), including 3 survey administrators, the project coordinator, and 2 study investigators. HCP, YPM, and study staff were asked a series of open-ended questions to learn about their experiences with the referral procedures, including any successful and challenging cases, and what (if anything) they would change about the safety protocol. These IDI were conducted in English by study team members with extensive training in qualitative methods and a comprehensive understanding of the project (KM, VB, and EA).

IDI were held in a private location in or near one of the clinics between January and April, 2019. IDI were audio-recorded, transcribed verbatim, and translated into English where needed. Interviewers wrote memos to self-reflect on and document their roles in the research process through reflexivity [34, 35] and to record their reflections on the interview guide, any methodological issues arising, and preliminary findings [35]. Memos were also used to document the research process, generating an “audit trail” to strengthen the dependability of findings [35].

### Analyses

Referral data were merged with baseline survey data to obtain the age and sex of those referred. Descriptive statistics were generated using counts and percentages. Differences in referral characteristics by youths’ sex were assessed using chi-square tests or Fisher’s exact tests for variables with small frequencies, and p values less than or equal to 0.05 were considered statistically significant. Thematic analysis was performed of coded transcripts in conjunction with written memos. The study team first developed a set of deductive codes based on the interview guides. One team member (KM) coded the transcripts, adding inductive codes iteratively based on emerging themes [36]. Coding was conducted with the support of NVivo 12. The study team jointly interpreted the quantitative and qualitative data, discussing findings extensively to refine interpretations. This involved consideration of points of convergence, divergence, contradictions, and relationships both within and between the methods [29]. Analyses were carried out in consultation with the Zambian research team members to support contextually-appropriate interpretation and enhance the credibility and confirmability of findings [35]. The goal was for “completeness,” [35] whereby the possibility of multiple realities would be recognized in an effort to generate a more nuanced understanding of the safety protocol’s implementation.

### Informed consent and ethics

Prior to enrollment in the RCT, study staff obtained informed consent, including consent to participate in qualitative IDI. In line with Zambian law, written parental/caregiver permission and youth assent was required for 15–17-year-olds [37]. The consent process for caregivers described the research in broad terms (e.g. health, safety) to protect minors who might be experiencing violence from the caregiver, while minors were provided greater detail, including that if they disclosed certain experiences (e.g., severe violence, suicidal ideation) they would be required to see a HCP. Youth aged 18–24, as legal adults, had the option of declining a HCP visit. Consent forms explained that YPM would be informed of any referrals made to a HCP (though not of the details) so that YPM could provide follow-up support. Informed consent for participating in an IDI was also obtained from HCP, YPM, and study staff.

Zambia’s Anti-Gender Based Violence (GBV) Act of 2011 requires medical practitioners to r eport situations of GBV [38]. This study did not trigger any mandatory reports. This research was reviewed and approved by the Zambia Ministry of Health through the National Health Research Authority, and the Johns Hopkins Bloomberg School of Public Health and Zambian ERES Converge ethical review boards

## Results

### Descriptive statistics

Almost half of youth enrolled in the RCT (45%, n = 124/276) were referred to a HCP at least once, including 41% (n = 46/111) of male and 48% (n = 78/165) of female youth; of these, 27 (22%) were referred on two separate occasions and 4 (n = 3%) on three separate occasions. Of the youth referred, about two-thirds were female and over a quarter were minors (ages 15–17 years) (Table 1). There were no statistically significant differences in characteristics of first referrals based on the youth’s sex. Three-quarters of referrals were made by survey interviewers and about 17% by YPM. The reason for the referral to a HCP was most often for sexual violence (35%), suicidal ideation/depression (29%), and physical violence (17%). Of those aged 18 and over, three quarters agreed to see a HCP, 7% declined to see a HCP but agreed to see the mental health nurse brought on by Project YES!, and 18% declined any referral. HCP referred 15% of youth participants externally for additional services.

**Table 1:** Characteristics of first referrals to a healthcare provider (n = 124 youth)

	Total	Male (n = 46)	Female (n = 78)	*p* value
Age				
15–17 years	35 (28.2%)	17 (37.0%)	18 (23.1%)	0.15
18–19 years	45 (36.3%)	17 (37.0%)	28 (35.9%)	
20–24 years	44 (35.5%)	12 (26.1%)	32 (41.0%)	
Role of person making referral				
Survey interviewer	93 (75.0%)	34 (73.9%)	59 (75.6%)	1.00*
Youth peer mentor	21 (16.9%)	8 (17.4%)	13 (16.7%)	
Other study staff	10 (8.1%)	4 (8.7%)	6 (7.7%)	
Reason(s) for referral to provider**				
Physical violence	26 (16.9%)	9 (15.8%)	17 (17.5%)	0.57*
Sexual violence	54 (35.1%)	22 (38.6%)	32 (33.0%)	
Emotional violence	10 (6.5%)	3 (5.3%)	7 (7.2%)	
Suicidal ideation/depression	44 (28.6%)	16 (28.1%)	28 (28.9%)	
Clinical/health issue	11 (7.1%)	2 (3.5%)	9 (9.3%)	
Food insecurity/hunger	1 (1.0%)	0 (0.0%)	1 (1.0%)	
Educational support	8 (5.2%)	5 (8.8%)	3 (3.1%)	
Referral to provider at clinic^				
Youth accepted referral	67 (75.3%)	21 (72.4%)	46 (76.7%)	0.92*
Youth declined referral but agreed to see the mental health nurse	6 (6.7%)	2 (6.9%)	4 (6.7%)	
Youth declined referral and declined to see the mental health nurse	16 (18.0%)	6 (20.7%)	10 (16.7%)	
Provider referred youth elsewhere^^				
No	92 (85.2%)	32 (80.0%)	60 (88.2%)	0.25
Yes	16 (14.8%)	8 (20.0%)	8 (11.8%)	
External referral location^*				
Children's hospital	6 (37.5%)	2 (25.0%)	4 (50.0%)	0.75*
Hospital psychosocial counseling center	3 (18.9%)	2 (25.0%)	1 (12.5%)	
Social welfare services	3 (18.9%)	2 (25.0%)	1 (12.5%)	
NGO for education	1 (6.3%)	1 (12.5%)	0 (0%)	
NGO for orphans and vulnerable children	1 (6.3%)	0 (0%)	1 (12.5%)	
Primary school	1 (6.3%)	1 (12.5%)	0 (0%)	
Gender-based violence one-stop center	1 (6.3%)	0 (0%)	1 (12.5%)	

*Denotes Fisher’s exact test. All other p values are from chi square tests

**Denominator is 154 reasons, since youth could be connected to a provider for multiple reasons at once

^Among those ages 18–24 (n = 89); youth ages 15–17 were minors and were required to see a provider

^^Among those who met with a provider (of any age group) (n = 108)

^*Among those referred elsewhere by a provider (n = 16)

NGO = non-governmental organization

### Qualitative findings

All 10 HCP, 8 YPM, 6 staff, and 29 out of 82 youth participants interviewed (20 female, 9 male) described their experiences with the safety protocol. Four key themes came out of the IDI.

#### Theme 1: Youths’ appreciation for their meetings with HCP

As the most salient theme from the youth IDI, youth welcomed the opportunity to meet with HCP through the safety protocol. Both male and female youth participants highlighted the benefits of speaking openly with a trained professional about the issues they were facing, which made them feel “unburdened” and “relieved.” For example:

The counselor asked me to be free and I told her everything and she advised me on the way forward. Thereafter, I felt OK…I could handle what was happening…I talked to the counselor for an hour plus. She asked me some questions which I answered and some questions I did not answer, and she understood…She gave me advice…I was open. I was free with her. (Male participant, aged 18 years)

I had the feeling that now I have someone to share my problems with. (Female participant, aged 23 years)

Some youth also described feeling “encouraged” by their meetings, which helped them gain clarity on the way forward. As one youth explained:

I was grateful because what [the healthcare provider] explained to me gave me a lot of knowledge….She even told me, ‘You have a long way to go…More opportunities are coming your way, so don’t think negatively’…I felt it helped. She told me, ‘If you have any problem, you should be coming to see me.’ (Female participant, aged 18 years)

Several said they planned to see a HCP again if another issue were to arise in the future.

#### Theme 2: Youths’ concerns about HCP referrals

Some youth expressed discomfort with being referred to a HCP, which was a less prominent but important theme in the youth IDI. Of the few participants who explained their reasons for declining the initial HCP referral, most were afraid that their situation would be shared with others at the clinic without their consent. For instance:

I never went to see the healthcare provider…because…maybe the nurse would tell other people, like the other nurses…[I have] a friend. She is a nurse, and she kind of tells us stories. (Female participant, aged 24 years)

One participant was “not comfortable” meeting with a HCP given her previous experience with a HCP sharing her HIV status with others at the clinic. A few participants described initially being hesitant but recognizing the benefits after seeing the HCP. A young man explained his transition in viewpoint as follows:

My reaction to the appointment I was given [is that] I was just going to get shouted at. I thought they would say bad things on me, but I realized they just wanted to advise and also encourage me. (Male participant, aged 22 years)

#### Theme 3: Referrals as a tool for strengthening HCP support for youth

As in the youth participant IDI, the most prominent theme in HCP, YPM, and study staff IDI concerned the benefits of carving out protected time for youth to meet with HCP about their problems. In IDI with HCP, the predominant viewpoint was that the safety protocol had helped HCP to better understand their youth clients’ situations. They came to recognize the many challenges facing youth living with HIV, their unique needs, and the importance of probing—or as one HCP explained, asking “a lot of questions so that you get to understand where they are, how they’re feeling, what problems they’re going through.” In turn, HCP were able to integrate support for ART adherence with interrelated issues, such as violence and suicidal thoughts. A HCP summarized her change in perspective as: “It’s more like we are putting everything in one package to help these adolescents who are living with HIV.”

The main theme in YPM and study staff IDI was that the safety protocol had helped many youth receive services by offering a protected time for HCP-youth interaction. Standard clinical review meetings do not typically provide a sufficient opportunity to tackle the “burdens” these youth face. YPM and survey interviewers appreciated the protocol’s design since they were themselves not equipped as counselors: “Once I [connect] them, they will get relevant attention and resources. The healthcare provider knows better of how to come up with a solution,” said one YPM. Several staff also noted the value of documenting whether youth had completed an external referral, since this feedback loop in standard care is typically missing.

#### Theme 4: Implementation challenges

Despite positive experiences with the safety protocol on the whole, a common and notable thread across the HCP, YPM, and study staff IDI concerned the challenges that emerged during implementation. These challenges in their order of salience included: (a) youth choosing not to open up to HCP, (b) time and resource constraints, (c) HCP training issues, and (d) stigma and cultural norms.

##### Youth choosing not to open up

HCP, YPM, and study staff explained that some youth chose not to share with a HCP the experiences they reported on a survey or discussed with their YPM. As a staff member said, “You’ll find that when you get to the healthcare provider, this particular client will show ignorance as though they did not respond ‘Yes’ to some of the [survey] questions.” This reluctance to share hindered the HCP’s ability to offer the youth support or connect them with other services. It also required additional time from the HCP to develop a full picture of the youth’s story. As a HCP explained, “Some youth didn’t say much. They didn’t want to share…You often have to work to get the young person to tell you what the problem is. The healthcare provider has to probe a lot.*”*

According to YPM and study staff, youth were concerned that HCP might share their experiences with others at the clinic. They also noted that some youth feared speaking openly since the HCP was “like a mother to them” and “they were afraid to be judged.” As a study staff member elaborated:

Some of them would refuse because there was some familiarity with the healthcare providers at the centers. Because maybe the healthcare providers have seen them grow and then maybe they tell them no sex before marriage…It will be difficult to go to the same provider. (Study staff member)

YPM and study staff felt that bringing on a mental health nurse part-way through the study was valuable in allowing the youth to meet with a counselor independent from their clinic. Even though a small proportion of the youth ultimately agreed to meet with the mental health nurse, YPM and study staff received positive feedback from these youth about their interactions with the nurse.

##### Time and resource constraints

HCP themselves, in addition to YPM and study staff, noted their tendency to be “overwhelmed” with their “work overload,” hindering their ability to prioritize Project YES! youth. Wait times with clients were sometimes very long, and if a HCP was unavailable, the clinic would reschedule the client for another day—except in emergencies. The nature and duration of referral meetings lacked consistency across clinics and providers. A few HCP were highlighted across IDI as having provided extensive support during the meetings. As one of these HCP explained, “Each time you have a session with this client, you establish a relationship which you cannot just get rid of. It becomes a lifetime relationship.” However, many HCP only met with a youth once about a given issue and did not formally schedule follow-ups, though they encouraged the youth to come see them again. Several study staff members expressed concerns about the quality and longevity of counseling provided. For example:

Healthcare providers were mostly busy …The participants, when you refer them, I thought they were not given much attention…Most of the referrals they met, it was just for a short period of time and usually the healthcare provider would be like, ‘She's OK,’ ‘We are done.’… There were very few [youth] who had continuous referral…. Some participants still had the same issues [during the 6 and 12 month visits]…as reported at baseline. (Study staff member)

These challenges were exacerbated by HCP turnover at the clinics. It took time for the new HCP to become accustomed to the safety protocol alongside other responsibilities.

##### Healthcare provider training

Some study staff described recognizing over the course of the study that HCP were not trained in handling all cases they encountered (e.g. mental health, educational support) despite being the first point of connection in the safety protocol. A staff member described the dilemma as follows:

[One healthcare provider] failed to handle an issue and then she told the youth peer mentor, ‘No, when you see these cases, don’t bring them straight to me, just take them to the psychiatric hospital’….In a way, she’s not wrong because she knows she can’t handle this. But then in our program, she is the primary connection. (Study staff member)

HCP themselves expressed feeling “powerless” in certain situations and wanted a greater understanding of community resources where they could refer youth when the issue was beyond their capacity. One HCP, for instance, described struggling with not knowing how best to handle a case; she said the “biggest issue…itching on me was the enablement to give them consistency of help…a pathway to further access help.”

##### Stigma and cultural norms

As a less prominent theme, stigma and cultural norms were raised as influencing the extent to which youth participants could receive the support they needed for violence and mental health problems. One HCP, for instance, described not wanting to refer youth externally for psychiatric services due to stigma associated with the uptake of these services. She anticipated that youth would turn down the referral “because they don’t want to be identified as crazy, nuts…” A study staff member described how HCP may have been less likely to refer youth elsewhere for services (e.g., to a GBV center) if dealing with emotional rather than physical or sexual violence, given cultural perceptions of what constitutes “violence.” He highlighted the need to strengthen HCP training on emotional forms of violence and mental health broadly to ensure that youth experiencing these challenges would universally receive the help they need:

Personal upbringing, culture…could affect the interpretation of…what is violence…Usually, I will think that healthcare providers may probably refer someone if they think the form of violence is severe…I think that healthcare providers may not be likely to refer a patient for services arising from emotional trauma…as opposed to the way they will refer somebody if they have physical injuries. I think that we need to strengthen and capacity-build ourselves to deal with emotional and mental health issues pertaining to violence. (Study staff member)

## Discussion

This study offers unique and practical insights into the successes and challenges of integrating a responsive safety system into a study measuring violence victimization and suicidal ideation among youth living with HIV in a SSA setting. Both male and female youth participants, alongside HCP, YPM, and study staff, described the protocol’s added-value of helping youth connect with HCP to talk through issues they are not typically able to discuss. The protocol helped HCP recognize the importance of asking youth questions to more deeply understand the intersectional issues they face. Implementation challenges included: youths’ concerns with opening up to HCP; constraints in time, resources, and training among clinic staff to support youth-HCP meetings; and stigma and cultural norms inhibiting external referrals for emotional trauma and mental health issues.

These findings contribute to the limited published experiences of safety protocols in low-resource settings [19, 20, 30] and should inform researchers’ safety planning—particularly when working with youth who are living with HIV. Although the safety protocol for this study was designed based on the resources and infrastructure of the study setting, the challenges raised in the IDIs echo findings from other studies in SSA on the importance of: (a) strengthening referral networks and coordination across services [19, 20, 39, 40]; (b) increasing the capacity of clinic providers to respond to a range of violence and mental health issues facing youth [40, 41]; (c) tackling broader contextual factors at play, such as cultural norms that minimize experiences of emotional violence and stigma around mental health [41]; and (d) encouraging an integrated approach to care for youth at HIV clinics [39]. Researchers working in similar settings should be aware of these issues when crafting their safety protocols and remain open to adapting their protocols, as we did in this study. Our findings also underscore the ethical responsibility of the research team to carefully plan for participant safety. This may require researchers to make difficult decisions, for instance, around whether to hire a counselor or social worker for the study or whether to work within the existing infrastructure [19, 21].

Our findings highlight the critical need to address violence and mental health issues among youth living with HIV, as almost half of both male and female youth were referred to a HCP for severe violence and/or suicidal ideation. Evidence is building on the links between experiences of physical, sexual, and emotional violence, as well as depression/suicidal thoughts, and virologic failure among youth in SSA [7, 10, 42]. The fact that these youth are in care represents an important opportunity to tackle the violence and mental health “burdens” they face for their personal wellbeing and to reach UNAIDS’ HIV target of 90% of those on treatment having suppressed viral loads [43]

These findings have implications for addressing experiences of violence among youth who attend HIV clinics in Zambia. Zambia’s legal and policy environment provides fertile ground for expanding violence prevention and response efforts to youth living with HIV. The government passed one of the most comprehensive gender-based violence laws in SSA [44], runs a large network of one-stop-centers for GBV [45], and has led and supported large GBV-prevention initiatives [46–48]. While these efforts indicate the seriousness with which the government aims to address rates of violence, our findings reinforce literature that HCP in Zambia work in resource-limited systems and referral structures in place are minimal [49, 50]. Even though our study protocol helped HCP recognize the importance of tackling violence, suicidal ideation, and adherence as “one package,” they struggle to meet these overwhelming needs. Although sexual violence was the leading reason for a referral, only one youth was referred to a GBV one-stop center. Moreover, efforts to address violence in Zambia tend to center on women and girls [51]. This focus is indeed critical given the persistence of gender inequality [51], but our findings also highlight a need to address experiences of violence against adolescent boys and young men living with HIV. We found no statistical differences by sex among those brought to a HCP for violence, building on our previous findings of a relationship between past-year violence victimization and virologic failure among both male and female youth in this study population [10].

This study fills an important gap in the literature on implementing safety protocols with vulnerable populations—in this case, youth living with HIV. A study strength is its use of both quantitative methods to describe referral characteristics and qualitative methods to gain perspectives from a variety of stakeholders, including the target population (i.e., youth) and implementers (i.e., HCP, YPM, and study staff). Given the sensitive nature of the topics of violence and mental health being discussed, participants may not have fully opened up to interviewers about their experiences with the safety protocol. Furthermore, social desirability bias may have been present in HCP, YPM, and study staff IDI given their roles in implementing the safety protocol, although this bias is likely to have been counterbalanced by the quantitative process data collected.

## Conclusions

I integrating a safety protocol into a clinic-based study addressing violence against youth living with HIV is possible and beneficial to youth and HCP alike. However, youths’ concerns about HCP referrals and gaps in resources and HCP capacity to handle all types of referrals may pose implementation challenges. These findings should inform the development of safety plans in future studies within low-resource settings, as researchers seek to fulfill their ethical obligations. Youth living with HIV need violence and mental health services and referrals, and the fact that they are accessing care at HIV clinics offers a critical entryway to meeting these needs. Innovative strategies must be developed to enhance HCP training, expand available resources, and strengthen referral systems within HIV clinics in Zambia.

## Supplementary Information

**Additional file 1**. Description of the Project YES! Safety Protocol.

## Data Availability

Data are available under Project SOAR’s subsection of the Harvard Dataverse: https://dataverse.harvard.edu/dataverse/projectsoar.
