# Supplementary material for: Experiences with a violence and mental health safety protocol for a randomized controlled trial to support youth living with HIV
Source: Glob Health Res Policy. 2021 Oct 15;6:40. doi: 10.1186/s41256-021-00224-0 (PMC8518229; doi:10.1186/s41256-021-00224-0)
Supplement: Supplementary file 1 — Additional file 1. Description of the Project YES! Safety Protocol. [file 41256_2021_224_MOESM1_ESM.docx]

**Experiences with a violence and mental health safety protocol for a randomized controlled trial to support youth living with HIV**

**Additional File 1: Description of the Project YES! Safety Protocol**

The safety protocol provided step-by-step guidance for addressing reports of violence and/or suicidal ideation from youth participants throughout the randomized controlled trial (RCT). Participants responding positively to the following survey items were “flagged”, i.e. the survey interviewer was notified by Magpi software of the need to bring the participant to a healthcare provider (HCP): a) any experience of severe physical violence in the past year (3 items); b) any experience of sexual violence ever (4 items); or c) thoughts of ending one’s life in the past week (1 item) (Table 1). Measures of violence, including definitions of severe physical and sexual violence, were drawn from the WHO Multi-Country Study on Women’s Health and Domestic Violence^51^ and the IPSCAN Child Abuse Screening Tool- Child Institutional (ICAST-CI).^52^ Participants were considered “high priority” cases if they reported severe physical or sexual violence in the past month (determined via a follow-up question prompted by Magpi) or suicidal ideation in the past week on the survey. Participants were also brought to HCP if youth peer mentors (YPM) or study staff had concerns about their wellbeing or if they expressed interest in seeing a HCP. Although surveys asked about moderate forms of physical violence (e.g. slapping, pushing) and psychological abuse (e.g. insulting, belittling), it was agreed that reports of these acts would not prompt a visit to a HCP unless requested by the participant.

**Table 1:** Survey measures prompting a visit to a healthcare provider

| **Type of measure** | **Questionnaire items** |
| --- | --- |
| Severe physical violence | Has anyone in the past year…1) Kicked you, dragged you, or severely beaten you up? 2) Choked you or burnt you on purpose? 3) Threatened to use or actually used a sharp object or other weapon against you? |
| Sexual violence | Has anyone ever…1) Made you watch a sex video or look at sexual pictures? 2) Made you look at their private parts or wanted to look at yours; 3) Touched your private parts in a sexual way, or made you touch theirs; 4) Physically forced you to have sexual intercourse when you did not want to? |
| Suicidal thoughts | In the past week, have you had thoughts about ending your life? |

When an issue was identified, the YPM or study staff member would complete a referral form before walking the participant to the designated HCP at the clinic and waiting with the participant until the HCP was ready. The HCP signed the referral form to confirm that the connection had been made. If the designated HCP was unavailable, the YPM/study staff requested to speak with the “back-up” designated HCP or the deputy in charge of the clinic. If unable to reach any provider, the YPM/study staff contacted the project coordinator for support with making the connection at the HCP’s earliest convenience for low-priority cases and as soon as possible for high-priority cases. Any follow-up with the participant by phone or in person was conducted by the team member who made the original connection. HCP handled cases according to clinical practice, local policy, and Zambian law. Where appropriate, HCP referred participants for services outside of the clinic. The project coordinator managed the safety procedures and offered daily in-person support to study staff and YPM and weekly in-person support to HCP. Safety protocol issues were also addressed during weekly calls between the study PIs and management team.

Referral forms tracked the date, name and role of person referring, clinic, activity during which the referral occurred (e.g., baseline survey, youth group meeting), reason(s) for referral (e.g., physical violence), and HCP to whom the participant was referred. The forms also tracked the progression of the case, including the dates of follow-up, notes about action taken, and external referrals made by the HCP (e.g. date, location referred to, etc.). Tracking continued through the study’s duration and for two weeks afterward to complete documentation. In response to a HCP request before the study’s launch, HCP were given a Project YES! notebook to record notes about meetings held and external referrals made. Referral forms and HCP notebooks were stored under lock and key at each clinic.

Three months into the project, participants who declined to see the HCP at their clinic were given the option of meeting a mental health nurse, who was independent of the four HIV clinics. The Project YES! team arranged transport to the mental health nurse for interested participants and the YPM/study staff member making the referral. Throughout the project, where a designated HCP transitioned out of his/her role at the clinic, the Project YES! team on-boarded an alternative HCP.
